# Blood lipid profile changes in type 2 diabetic rats after tail suspension and reloading

**DOI:** 10.1186/s12944-021-01511-y

**Published:** 2021-08-01

**Authors:** Shoji Tanaka, Sachiko Madokoro, Pleiades Tiharu Inaoka, Toshiaki Yamazaki

**Affiliations:** Department of Rehabilitation, Faculty of Health Sciences, Institute of Medical, Pharmaceutical and Health Sciences, Kanazawa University, 5-11-80 Kodatsuno, Kanazawa, 920-0942 Japan

**Keywords:** Blood lipid profile, Non-high-density lipoprotein cholesterol, Tail suspension, Type-2 diabetes mellitus, Reloading

## Abstract

**Purpose:**

The effects of the tail suspension and reloading on the protein and lipid metabolism in muscle and blood in type 2 diabetes mellitus (T2DM) are unclear. This study evaluated the hypothesis that skeletal muscle catabolism is greater in T2DM than in non-diabetes mellitus (non-DM) rats and that the activity-dependent changes in the intramuscular lipid accumulation and blood lipid profile are poorer in T2DM than in non-DM rats.

**Methods:**

T2DM and non-DM rats were suspended for two weeks followed by reloading for two weeks. The muscle and blood were then examined.

**Results:**

In contrast to our hypothesis, there was no marked difference between the T2DM and non-DM groups in terms of the skeletal muscle catabolism and activity-dependent changes in intramuscular lipid accumulation. However, the blood lipid profile increased in the T2DM group compared to the non-DM group. One interesting finding in this study was the decrease in non-high-density lipoprotein (non-HDL) cholesterol levels after one week of reloading followed by a significant increase in the non-HDL cholesterol levels after two weeks of reloading in the T2DM group.

**Conclusion:**

These results suggest that a dramatic increase in activity after a period of inactivity may rapidly improve the blood lipid profile in T2DM rats.

**Supplementary Information:**

The online version contains supplementary material available at 10.1186/s12944-021-01511-y.

## Introduction

Diabetes can reduce the healthy life expectancy in an aging society. Although the relationship between diabetes and skeletal muscle protein metabolism has not been fully elucidated, it has been pointed out that an increase in the blood glucose level, which is the main symptom of diabetes, causes a decrease in skeletal muscle amount due to an increase in muscle protein degradation induced by hyperglycemia [1]. Lifestyle optimization has thus been shown to play an important role in improving diabetes [2, 3].

In people with T2DM and obesity, the accumulation of ectopic lipid droplets is observed in some cells, such as skeletal muscle [4]. While this ectopic lipid droplet retention is highly correlated with T2DM and insulin resistance, lipid droplet accumulation is also observed in athletes’ skeletal muscle cells. This contradictory phenomenon is called the athlete paradox [5]. Intramuscular lipid droplets (IMLDs) have been reported to increase with chronic training [6], but the effects of physical activity on IMLDs in T2DM are still unclear.

The diagnosis of metabolic syndrome predicts cardiovascular disease and T2DM [7]. The diagnostic criteria for metabolic syndrome are an increased abdominal circumference, hypertriglyceridemia, low high-density lipoprotein (HDL) cholesterol (cho), high blood pressure, and fasting hyperglycemia [8]. Decreased triglycerides, low-density lipoprotein (LDL) cho, and non-HDL cho levels reduce the risk of cardiovascular disease following metabolic syndrome [9]. Improvement in one’s lifestyle improves the total and HDL cho levels and thereby the risk of metabolic syndrome [10]. Thus, one’s physical activity status is closely related to the development of T2DM.

Regardless of the presence or absence of diabetes, there may be situations in which an accident or illness imposes activity restrictions, followed by repeated physical activity restrictions and eventually re-activity when returning to daily life. Therefore, for rehabilitation, it is important to determine whether or not the physical activity-dependent protein and lipid metabolism differs between healthy and diabetic subjects in order to predict the prognosis and determine the effect of intervention. However, it is ethically difficult to conduct studies on people with T2DM that induce physical limitations followed by physical activity reactivation. Also, there are limitations regarding the reproduction of similar characteristics in relation to daily activity and to match similar profiles in humans. No conclusions have been reached on the debate over whether exercise improves the blood lipid profile of individuals with T2DM in comparison to those without diabetes mellitus (non-DM).

Thus far, in studies using T2DM animals prepared by the administration of alloxan, the blood lipid profile could not be improved by forced swimming at 3.5% load of body weight (BW) [11], however, it could be improved by forced swimming at 90% of the maximal lactate steady state [12]. Ribeiro [13] stated that physical training at high intensity is important for obtaining better blood lipid profile changes. Despite these reports, no reports have so far investigated the effects of long-term physical activity restriction (tail suspension) and re-activity (reloading) on intramuscular lipid accumulation and the blood lipid profiles in non-DM and T2DM groups.

The present study therefore evaluated the hypothesis that skeletal muscle catabolism is greater in T2DM than in non-DM rats, and the activity-dependent changes in intramuscular lipid accumulation and blood lipid profile are poorer in T2DM than in non-DM rats.

## Materials and methods

### Animals

Eight-week-old male Wistar rats (BW: 193.6 ± 1.4 g) and Goto-Kakizaki (GK) rats (BW: 211.1 ± 1.4 g), T2DM model animals originating from Wistar rats, were purchased from Kiwa Laboratory Animals (Wakayama, Japan) and housed at 20–24 °C on a 12-h light/dark cycle with free access to food and water. Wistar rats and GK rats were both divided into a non-DM group and T2DM group, respectively. Each rat was assigned to either the control (CON) group (*n* = 8 for each) or tail suspension (SUS) group (n = 8 for each) for 14 days, a group receiving reloading for 1 week following tail suspension (R1w, n = 8 for each), and a group receiving reloading for 2 weeks following tail suspension (R1w, n = 8 for each).

The method of Morey-Holton [14] was used to apply tail suspension, causing atrophy of the hindlimb muscles. Suspended rats were fed a standard pelleted chow and HydroGel^®^ (EP Trading, Tokyo, Japan; 98% pure water) for hydration.

The CON group was bred for the same term as the SUS group. After the breeding period, all rats were deeply anesthetized with 2% isoflurane, and the gastrocnemius (Gas) muscles from both hindlimbs as well as the blood (2–2.5 mL) were collected. All rats were fasted for 10 h before blood sampling (before tail suspension and tissue extraction). The lateral and medial Gas muscle from the left hindlimb was dissected, and the wet weight was measured. After wet weight measurement, the muscles were soaked in ethanol for 3 days, followed by drying at 40 °C for 3 days, and then the muscle dry weight was measured. The muscle wet weight per BW, muscle dry weight per BW, and muscle dry weight per muscle wet weight were calculated to determine the muscle mass, muscle component protein (MCP) mass, and MCP ratio, respectively. The central region of the lateral Gas muscle from the right hindlimb was quickly frozen with liquid nitrogen, and the medial Gas muscle of the right hindlimb was placed in Tissue-Tek optimal cutting temperature compound (Sakura Finetek Japan, Tokyo, Japan) and quickly frozen in isopentane cooled by liquid nitrogen. The frozen tissues were stored at − 80 °C until further use. The blood was left to sit for at least 30 min and then centrifuged at 4400 rpm. From each blood sample, serum was isolated and analyzed by SRL Inc. (Tokyo, Japan). The fasting blood glucose levels were measured using a blood glucose self-monitoring device (Care Fast^®^ R; Nipro, Osaka, Japan) before and after tail suspension and during tissue sampling.

The experimental procedures were approved by the Animal Welfare Committee of Kanazawa University.

### Intramuscular lipid droplets in the whole muscle

Lipid infiltration was analyzed as described by Blitz [15]. The lateral Gas muscle of the right hindlimb was soaked in 1% solution of sodium dodecyl sulfate (SDS; Sigma-Aldrich Japan, Tokyo, Japan) in phosphate-buffered saline (PBS) with agitation at room temperture (RT) for 10 days. The muscle was fixed in 4% paraformaldehyde at RT for 5 days, washed 3 times with PBS, and then incubated in 60% isopropanol with saturated oil red O (ORO; Fujifilm Wako Pure Chemical, Osaka, Japan) for 120 min with agitation. The muscle was washed overnight in 1% SDS in PBS with agitation to remove unbound ORO and then soaked in a 3-fold volume of isopropanol for 30 min with agitation while lipid-bound ORO was eluted from the whole muscle. The eluted ORO concentration was measured by a spectrophotometer (Titec, Saitama, Japan) at 500 nm. The ORO concentration values were normalized with isopropanol at 500 nm and presented as IMLDs.

### Muscle damage and lipid droplets in muscle sections

The frozen medial Gas muscle of the right hindlimb was cut into 10-μm-thick transverse sections using a CM-41 cryostat (Sakura Finetek Japan) and air-dried for 10 min at RT. Cryo-sections were subjected to hematoxylin and eosin and ORO staining. ORO staining of sections was performed according to Mehlem [16]. Each section was incubated in 60% isopropanol with saturated ORO, washed in distilled water, and then mounted with water-soluble mounting medium (Nichirei Biosciences, Tokyo, Japan). The sections were visualized using a microscope (BX50; Olympus, Tokyo, Japan).

### Serum profile

Serum total protein, albumin and triglyceride were determined by Biuret, Bromocresol green, GPO-HMMPS, respectively, while total cho and creatinine were determined by an enzyme reaction method using an automatic biochemistry analyzer (Hitachi 7180, Hitachi, Tokyo, Japan). HDL and LDL cho were determined by a direct method using an automatic analyzer (BioMajesty™ JCA-BM8000 series, Japan Electron Optics Laboratory, Tokyo, Japan).

### Statistical analyses

Data are presented as the mean ± standard error. Statistical significance was evaluated using a two-way analysis of variance (ANOVA) following Bonferroni’s test or Tukey’s post-hoc test. Bonferroni’s test was used for the following comparisons: “CON *vs*. SUS *vs.* R1w *vs.* R2w in non-DM”, “CON *vs.* SUS *vs.* R1w *vs.* R2w in T2DM” and “non-DM *vs.* T2DM of CON, non-DM *vs.* T2DM of SUS, non-DM *vs.* T2DM of R1w and non-DM *vs.* T2DM of R2w”, when there was an interaction after the two-way ANOVA. Tukey’s post-hoc test was used to compare “CON *vs.* SUS *vs.* R1w *vs.* R2w”, when there was no interaction, however a simple main effect was observed after intervention. All statistical analyses were performed using the SPSS software program (v18.0.0). *P* values of < 0.05 were considered to indicate statistical significance.

## Results

### BW and muscle weight

**Fig. 1:**
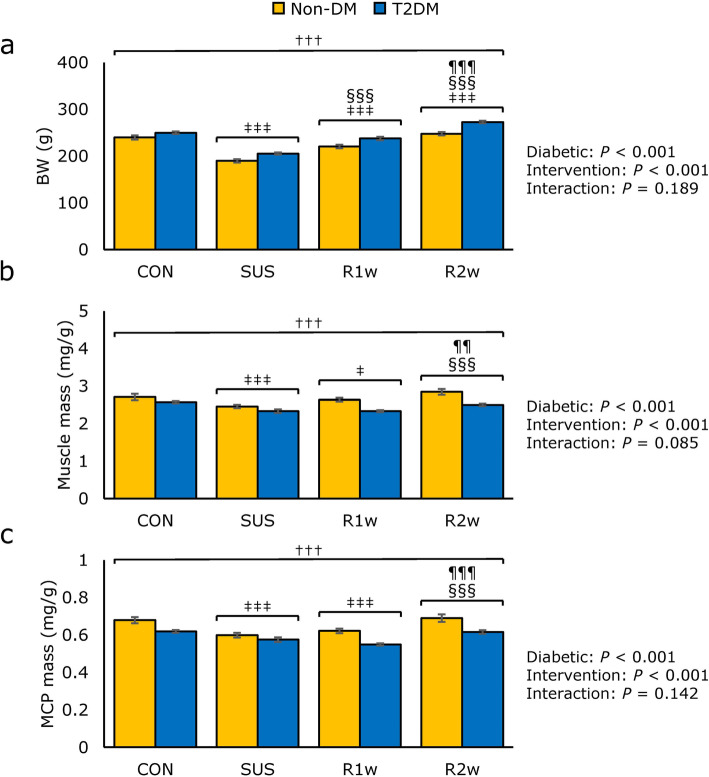
Body weight, muscle mass, and muscle component protein (MCP) comparisons. Comparisons of the body weight (BW) (**a**), muscle wet weight per BW (Muscle mass) (**b**), and muscle dry weight per BW (MCP mass) (**c**) among the control group (CON), tail suspension group (SUS), one-week reloading following tail suspension group (R1w), and two-week reloading following tail suspension group (R2w) in non-diabetes mellitus (non-DM) rats (yellow column) and type 2 diabetes mellitus (T2DM) rats (blue column). Statistical symbols: ^†††^: *P* < 0.001 non-DM vs. T2DM; ^‡^ *P* < 0.05 and ^‡‡‡^: *P* < 0.001 vs. CON; ^§§§^: *P* < 0.001 vs. SUS; ^¶¶^: *P* < 0.01 and ^¶¶¶^: *P* < 0.001 vs. R1w

The BW, muscle mass, and MCP mass of the left lateral Gas muscle were measured to assess the changes in the skeletal muscle amount. To this end, two-way ANOVA with the diabetes and intervention groups (tail suspension, 1 week and 2 weeks of reloading) was performed as an independent variable and measurements as the dependent variable to examine whether or not there was a difference in the mean value of the evaluation factors between the diabetes and intervention groups (Fig. 1). The BW, muscle mass, and MCP mass of the animals were significantly different for both the main effect of the diabetic factors and the main effect of the intervention factors, but no interaction was found (Fig. 1a-c). Regarding the BW, a subsequent Tukey’s post-hoc test revealed that the BW was significantly higher in the T2DM group than in the non-DM group and was significantly different among all intervention factors (Fig. 1a). Regarding the muscle mass and MCP mass, Tukey’s post-hoc test revealed that the masses were significantly lower in the T2DM group than in the non-DM throughout the experimental period, and the SUS and R1w groups had significantly lower values than the CON and R2w groups (Fig. 1b and c). The values of the left medial Gas muscle are indicated in the supplemental material.

### MCP ratio and IMLD

**Fig. 2:**
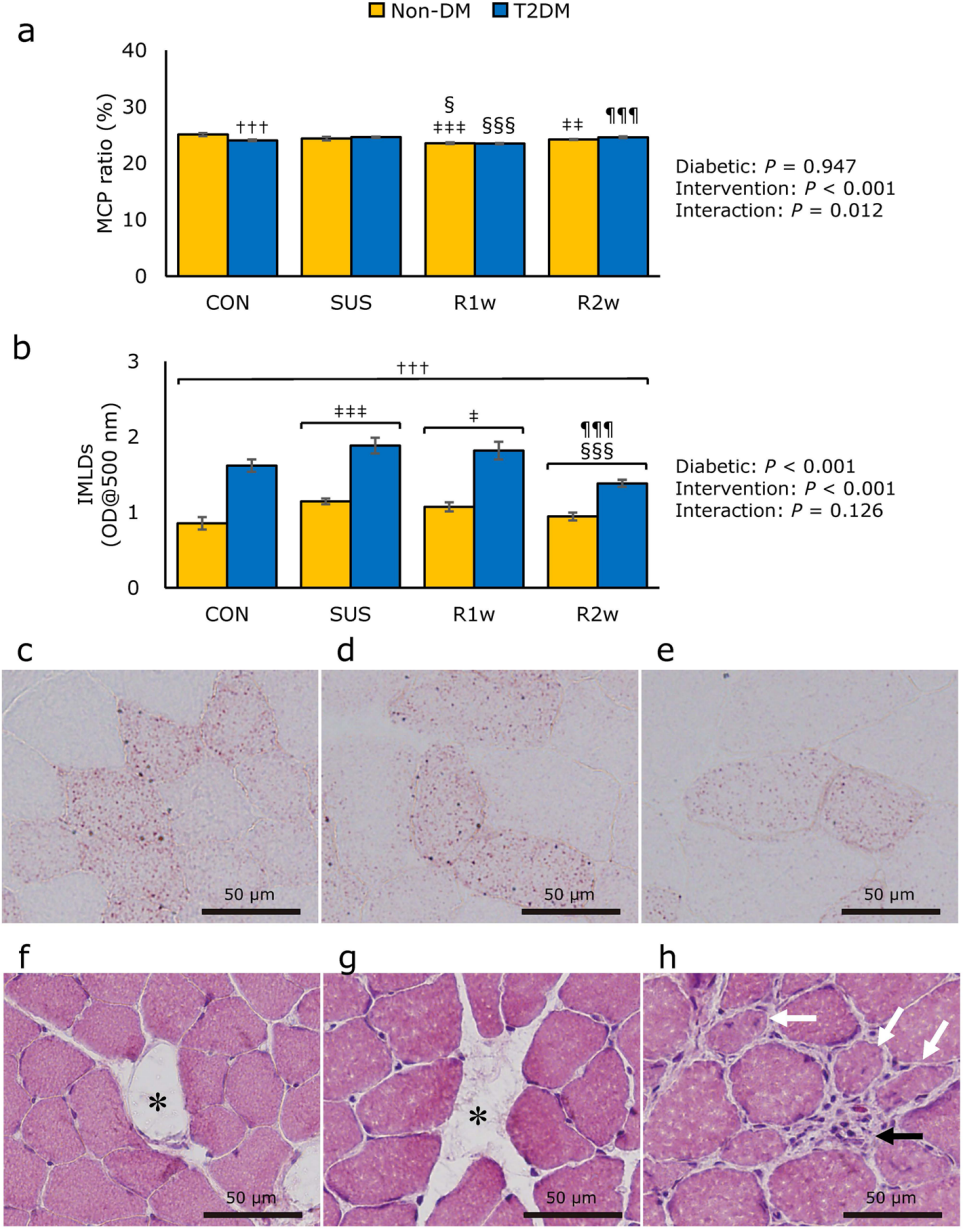
Composition of the muscle component protein, concentration of intramuscular lipid droplets, and muscle damage histological images. Comparisons of the muscle dry weight per wet weight (MCP) ratio (**a**) and concentration of intramuscular lipid droplets (IMLDs) (**b**) among the control group (CON), tail suspension group (SUS), one-week reloading following tail suspension group (R1w), and two-week reloading following tail suspension group (R2w) in non-diabetes mellitus (non-DM) rats (yellow column) and type 2 diabetes mellitus (T2DM) rats (blue column). IMLDs in cryo-sections of the right medial Gas muscle stained by oil red O staining after tail suspension (SUS) in the T2DM group (**c**) and two weeks after reloading (R2w) in the T2DM (**d**) and non-DM groups (**e**). Gastrocnemius cryosections stained by hematoxylin and eosin showing digested and absorbed myofibers (**f** and **g**, asterisks), central nucleated fibers (**h**, white arrows), and necrotic fibers infiltrated with inflammatory cells (**h**, black arrow) in the non-DM (**f**) and T2DM groups (**g** and **h**) after one week of reloading (R1w). Figure (**a**) statistic symbols: ^†††^: *P* < 0.001 vs. non-DM under the same conditions; ^‡‡^: *P* < 0.01 and ^‡‡‡^: *P* < 0.001 vs. CON; ^§^: *P* < 0.05 and ^§§§^: *P* < 0.001 vs. SUS of the same group (non-DM or T2DM); ^¶¶¶^: *P* < 0.001 vs. R1w of the same group. Figure (**b**) statistic symbols: ^†††^: *P* < 0.001 vs. non-DM; ^‡^: *P* < 0.05 and ^‡‡‡^: *P* < 0.001 vs. CON; ^§§§^: *P* < 0.001 vs. SUS; ^¶¶¶^: *P* < 0.001 vs. R1w

The MCP ratio was significantly different for both the main effect of the intervention factors and the interaction according to a two-way ANOVA (Fig. 2a). Subsequent Bonferroni’s test revealed that the MCP ratio was significantly lower in the T2DM group than in the non-DM at CON. In addition, the MCP ratio in the non-DM was shown to be significantly lower at R1w than at CON and SUS and lower at R2w than at CON, and the MCP in the T2DM group was significantly lower at R1w than at SUS and R2w.

The concentration of IMLDs was assessed using the entire right lateral Gas muscle. The concentration differed significantly among diabetic factors and intervention factors, but no interaction was noted on a two-way ANOVA (Fig. 2b). Subsequent Tukey’s post-hoc test revealed a significantly greater concentration of IMLDs in the T2DM group than in the non-DM group, and the concentrations in both the non-DM and T2DM groups at CON and R2w were significantly lower than those at SUS or R1w. The IMLDs were detected by ORO staining using cryo-sections from the right medial Gas muscle (Fig. 2c-e). The concentration of IMLDs was highest at SUS in the T2DM group (Fig. 2c) and then decreased significantly at R2w in both the T2DM group (Fig. 2d) and non-DM group (Fig. 2e).

### Muscle damage

The muscle damage induced by reloading was examined using the right medial Gas muscle one week after reloading in the non-DM (Fig. 2f) and T2DM groups (Fig. 2g-h). Digested and absorbed myofibers (Fig. 2f and g), necrosed fibers, myofibers infiltrated with inflammatory cells, and central nucleated fibers (Fig. 2h) were observed in the non-DM (Fig. 2f) and T2DM groups (Fig. 2g and h). The muscle injury pattern was scattered, as shown in these figures.

### Blood glucose levels

**Fig. 3:**
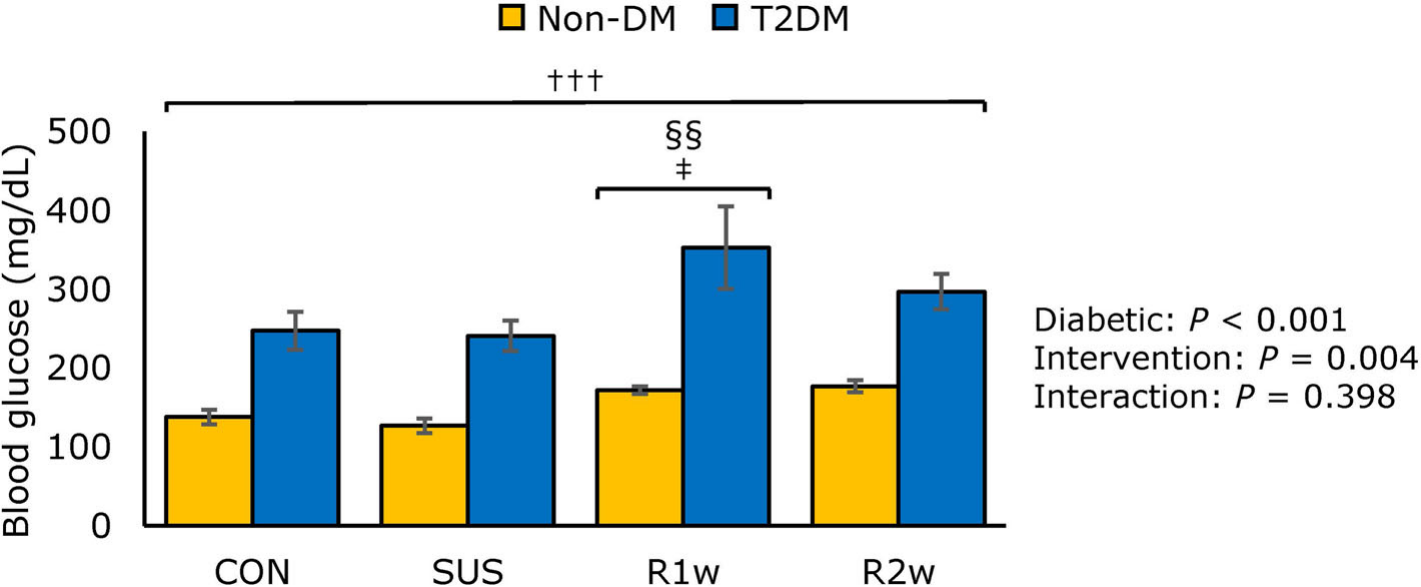
Blood glucose. Comparisons of the blood glucose levels among the control group (CON), tail suspension group (SUS), one-week reloading following tail suspension group (R1w), and two-week reloading following tail suspension group (R2w) in non-diabetes mellitus (non-DM) rats (yellow column) and type 2 diabetes mellitus (T2DM) rats (blue column). Statistical symbols: ^†††^: *P* < 0.001 non-DM vs. T2DM; ^‡^ *P* < 0.05 vs. CON; ^§§^: *P* < 0.01 vs. SUS

Respective blood glucose levels in the non-DM and T2DM groups were 113.5 ± 3.8 and 164.2 ± 5.3 mg/dL (6.31 ± 0.21 and 9.12 ± 0.29 mmol/L) before tail suspension. Two-way ANOVA revealed that the blood glucose levels in the T2DM group were significantly higher than in the non-DM group both before tail suspension and at each tissue collection point. The blood glucose levels at tissue collection differed significantly among diabetic factors and intervention factors according to a two-way ANOVA, but no interaction was found (Fig. 3). Subsequent Tukey’s post-hoc test revealed that the blood glucose levels in both groups were significantly higher at R1w than at CON or SUS.

### Serum profile

**Fig. 4:**
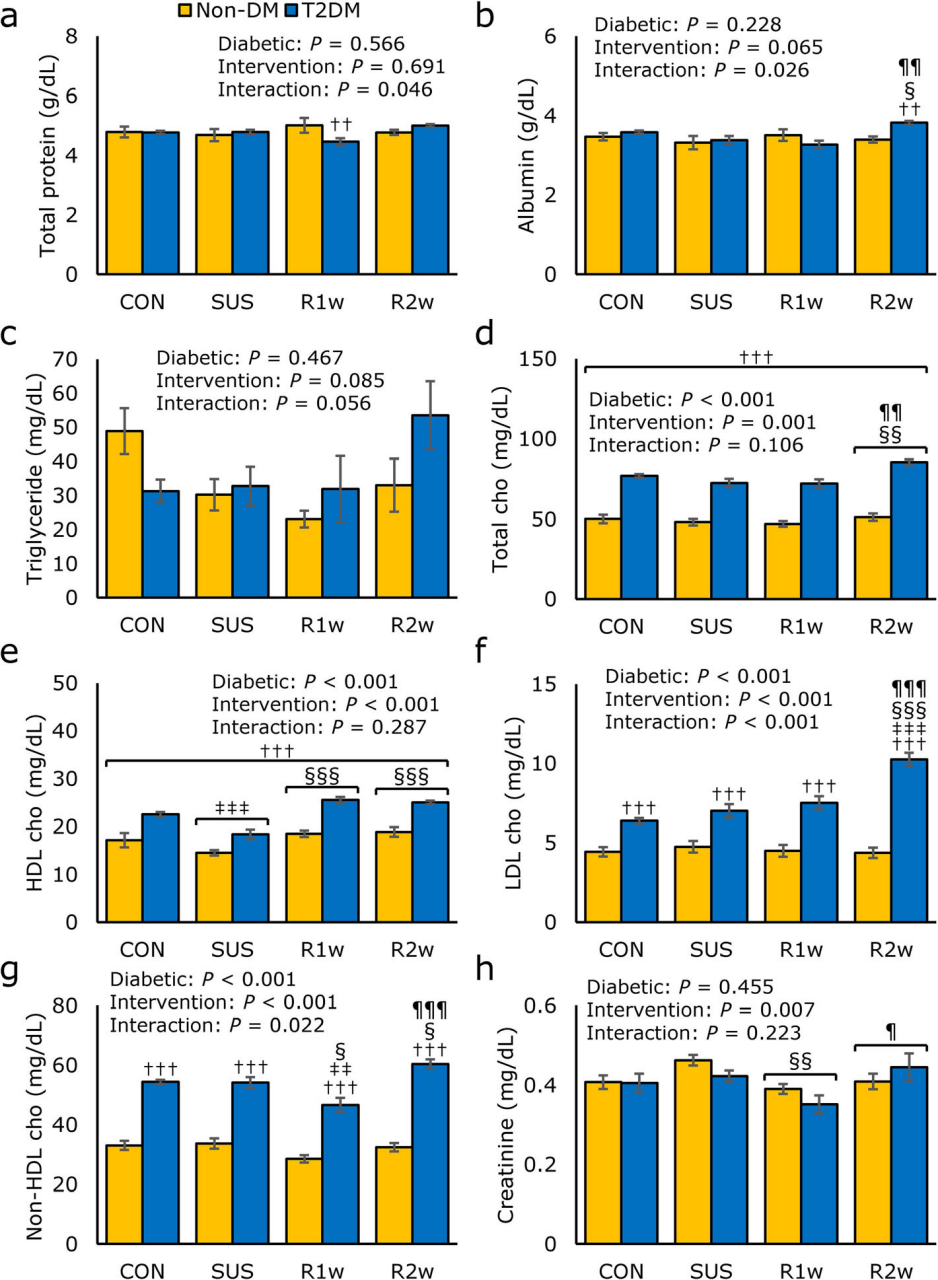
Serum profile comparisons. Comparisons of the serum levels of total protein (**a**), albumin (**b**), triglyceride (**c**), total cholesterol (cho) (**d**), high density lipoprotein (HDL) cho (**e**), low density lipoprotein (LDL) cho (**f**), non-HDL cho (**g**), and creatinine (**h**) in the control group (CON), tail suspension group (SUS), one-week reloading following tail suspension group (R1w), and two-week reloading following tail suspension group (R2w) in non-diabetes mellitus (non-DM) rats (yellow column) and type 2 diabetes mellitus (T2DM) rats (blue column). Statistical symbols (**a, b, f** and **g**): ^††^: *P* < 0.01 and ^†††^: *P* < 0.001 vs. non-DM under the same conditions; ^‡‡^: *P* < 0.01 and ^‡‡‡^: *P* < 0.001 vs. CON of same group (non-DM or T2DM); ^§^: *P* < 0.05 and ^§§§^: *P* < 0.001 vs. SUS of same group; ^¶¶^: *P* < 0.01 and ^¶¶¶^: *P* < 0.001 vs. R1w of same group. Statistical symbols (**d**, **e** and **h**): ^†††^: *P* < 0.001 vs. non-DM; ^‡‡‡^: *P* < 0.001 vs. CON; ^§§^: *P* < 0.01 and ^§§§^: *P* < 0.001 vs. SUS; ^¶¶^: *P* < 0.01 and ^¶¶¶^: *P* < 0.001 vs. R1w

The serum profile was assayed using blood collected immediately after tissue collection (Fig. 4). A two-way ANOVA revealed significant interaction for total protein (Fig. 4a). Subsequent Bonferroni’s test revealed that total protein levels were significantly lower in the T2DM group than in the non-DM group at R1w. A significant interaction was found for albumin (Fig. 4b). Subsequent Bonferroni’s test revealed that albumin levels were significantly higher in the T2DM group than in the non-DM group at R2w and higher than the T2DM values at SUS and R1. No significant difference in the triglyceride values were noted (Fig. 4c). Total cho levels differed significantly among diabetic factors and intervention factors, but no interaction was found (Fig. 4d), indicating that total cho levels were significantly higher in the T2DM group than in the non-DM group. Subsequent Tukey’s post-hoc test revealed that the total cho level was significantly higher at R2w than at SUS and R1w in both the non-DM and T2DM groups. HDL cho levels differed significantly among diabetic factors and intervention factors, but no interaction was found (Fig. 4e), indicating that HDL cho levels were significantly higher in the T2DM group than in the non-DM group. Subsequent Tukey’s post-hoc test revealed that the HDL cho levels were significantly lower at SUS than at CON, R1w, or R2w in both the non-DM and T2DM groups. The LDL cho levels differed significantly among diabetic factors and intervention factors, and interaction was found (Fig. 4f). Subsequent Bonferroni’s test revealed that the LDL levels were significantly higher in the T2DM group than in the non-DM group, and the values in the T2DM group at R2w were significantly higher than at other points. Non-HDL cho levels differed significantly among diabetic factors and intervention factors, and significant interaction was noted (Fig. 4g). Subsequent Bonferroni’s test revealed that non-HDL cho levels were significantly higher in the T2DM group than in the non-DM group, and the values in the T2DM group at R1w were significantly lower than those at other points, while the values at R2w were significantly higher than those at SUS and R1w. Creatinine levels differed significantly among intervention factors, but no interaction was found (Fig. 4h). Subsequent Tukey’s post-hot test revealed that the creatinine levels in both the non-DM and T2DM groups at R1w were significantly lower than those at SUS and R2w.

## Discussion

The present study evaluated the hypothesis that skeletal muscle catabolism is higher in T2DM than in non-DM rats, and that activity-dependent changes of intramuscular lipid droplet accumulation and blood lipid profile are poorer in T2DM than in non-DM rats. The results did not completely support the hypothesis, as there was no marked difference in the skeletal muscle protein level and activity-dependent changes of intramuscular lipid accumulation between non-DM and T2DM rats. However, some differences in activity-dependent changes were found in the blood lipid profile between the groups. Of note, the decrease in non-HDL cho levels after one week of reloading was followed by a significant increase in non-HDL cho levels after two weeks of reloading in the T2DM group.

Firstly, the muscular characteristics of GK rats and the effects of intervention (hypoactivity by tail suspension and re-activation by reloading) were examined. The GK rats used in this study were introduced as nonobese T2DM rats [17]. However, while the BW was higher in the T2DM group than in the non-DM group, the initial Gas muscle amount (muscle mass, MCP mass, and MCP ratio) was lower in the T2DM than in the non-DM group. The GK rats tended to be obese in this study. Yasuda [17] reported that the BW and muscle mass were low in T2DM rats. However, the present results differed from those of the previous report with regard to BW. The reason for this discrepancy is unclear, but it may be due to differences in laboratory animal acquisition routes.

Next, activity-dependent changes in the muscles were examined. The hypothesis that muscle has a stronger catabolism effect in T2DM than in non-DM was denied. The muscle mass and MCP mass decreased after two weeks of tail suspension and increased after two weeks of reloading. Previous studies reported that two-week tail suspension caused Gas muscle atrophy in rodents [18–21], and subsequent two-week reloading caused recovery of the Gas muscle wet weight in young adult mice [21] and rats [19]. In addition, a previous study of unloaded T2DM rats followed by a week of reloading revealed no marked difference in the course of soleus muscle mass loss and recovery [22]. Therefore, the results were consistent with those studies, indicating that muscle atrophy was caused by tail suspension. The MCP and IMLDs were examined to clarify the intramuscular accumulation during tail suspension and reloading. Muscle is mainly comprised of MCP, such as myofibrils, capillaries and peripheral nerves, lipids, and water. Therefore, the muscle dry weight was the amount of muscle remaining after the removal of water and lipids. Lipids were removed by soaking the muscles in ethanol after measuring the muscle wet weight. Changes in MCP ratios were found between tail suspension and one week of reloading in this study. The analysis of the MCP mass, MCP ratio, and concentration of IMLD in both periods suggests a relative increase in liquid but not in the protein or lipid content after one week of reloading. The increase in the MCP ratio after two-week reloading in this study suggests an increase in the muscle component protein and liquid content. Perry et al. [23] reported that skeletal muscle of T2DM patients showed increased levels of various pro-inflammatory cytokines, such as tumor necrosis factor α and interleukin 6, and the development of insulin resistance which are related to the decrease in the protein anabolism. The findings of this study suggest that the muscle in T2DM rats is fragile and prone to atrophy. Previous studies [24, 25] have also reported muscle damage after reloading, and in our present tissue analysis, the myofiber damages in the non-DM and T2DM groups occurred one week after reloading.

The insulin value was not measured in this study, so insulin resistance is unclear. Therefore, the cause of muscle loss at R1w in T2DM remains speculative, but given the high fasting blood glucose level at R1w, it may have been caused by insulin resistance or increased inflammation due to reloading. However, the level of muscle damage after reloading was not shown to differ markedly between non-DM and T2DM rats [22], and the anabolic response to diet was similar in T2DM subjects and age-matched controls [26]. Therefore, the findings were similar to previous results, suggesting that the muscle protein metabolism did not differ markedly due to activity-dependent changes between non-DM and T2DM rats.

The differences in IMLD concentration between the non-DM and T2DM groups in this study were consistent with previous findings of greater IMLD concentrations in T2DM patients and animals than in healthy controls [5, 27, 28]. It was also predicted that the activity-dependent changes in IMLDs would be poorer in the T2DM group than in the non-DM group, but this hypothesis was denied. A previous study found that IMLD levels were reduced by exercise training in T2DM patients [29]. However, this study showed no evidence that T2DM in rats influenced activity-dependent changes in IMLD concentrations. The positive influence of exercise [30, 31] and concomitant negative influence of insulin resistance [32] on the metabolism of IMLDs may underlie the difficulty of detecting activity-dependent changes in IMLDs.

Finally, the nutrition, lipid status, and renal function were estimated using serum. As a result, the hypothesis that the activity-dependent change in blood lipid profile was worse in T2DM than in non-DM rats was denied. However, there was a notable decrease in the non-HDL cho levels at one week of reloading followed by a significant increase in the non-HDL cho levels at 2 weeks after reloading in the T2DM group, thus suggesting that the T2DM blood lipid levels are more sensitive to activity changes. Nutritional changes in total protein at one week after reloading and in albumin at two weeks after reloading in the T2DM group. The serum total protein levels, including albumin, are influenced by the nutritional status; and/or by diseases that cause dyshepatia, such as hepatocirrhosis [33]. The albumin serum level can also decrease following protein-energy wasting with nephrotic syndrome [34] and it is frequently used as a nutritional status marker [35]. Albumin serum levels can be reduced by various diseases, whereas albumin can be increased by strenuous exercise or acute dehydration [36–38]. Serum levels of creatinine decrease due to muscle loss, early-stage diabetes, and diabetes insipidus and increase due to increased muscle mass or diabetic renal dysfunction. While the BW and creatinine levels did not differ significantly between the non-DM and T2DM groups, they were higher at R2w than at R1w. Therefore, although the activity status in the non-DM and T2DM groups was not measured, the high albumin levels at R2w in T2DM were probably not due to dehydration. Thus, those changes in the albumin and creatinine levels may have been influenced by muscle mass changes, as seen in the MCP ratio in this study. The variance in the levels of triglyceride, total cho, and HDL cho between the activity states were not markedly different between the non-DM and T2DM groups. The HDL cho level changed according to the activity level; decreased activity due to tail suspension decreased the level, whereas increased activity due to reloading increased the level, which was in agreement with the conventional understanding [39]. The present results also showed that the activity-dependent changes in HDL cho were unaffected by the presence of T2DM, which is supported by a meta-analysis [40] investigating the relationship between a T2DM lifestyle and HDL cho levels. LDL cho levels differed between the non-DM and T2DM groups in this study, and the T2DM rats showed increased LDL cho levels after two-week reloading. Previous meta-analyses [41, 42] reported that LDL cho levels are reduced by exercise, weight loss, and a reduction in trans fatty acid consumption, but the increased levels in the T2DM group after two weeks of reloading did not appear to be related to the physical activity. The cause of this increase in LDL cho is unknown, but potential overeating in this group cannot be excluded. The non-HDL cho levels in the T2DM group decreased after one-week reloading. Since non-HDL cho is the value obtained by subtracting the HDL cho from the total cho, it was concluded that the increase in total cho was smaller than the increase in HDL cho. Therefore, this result may indicate that, in the T2DM rats, the HDL cho ratio to the total cho ratio has one week of reloading. The results of this study were characterized by a decrease in non-HDL cho (increased HDL cho ratio) in the early stages of reloading in T2DM rats and a significant increase in LDL cho thereafter (two weeks of reloading). These results indicate that the blood lipid profiles of T2DM rats may demonstrate positive early changes after one week of reloading but negative changes after two weeks of reloading. It is pointed out that HDL cho may be a regulator of the inflammatory response in macrophages [43]. Bortolon [44] reported that the inflammatory response to intense exercise were greater in T2DM rats than in non-DM rats. Therefore, the acute elevation of HDL cho observed in the present study may be associated with muscle inflammation due to acute activity changes in T2DM.

### Study strengths and limitations

A high non-HDL cho concentration increases the risk of coronary heart disease [45]. Improving the lipid profile is an important aim in rehabilitation of T2DM patients. The acute elevation with re-activity in HDL cho in relation to total cho that was observed in our T2DM group may support the early efficacy of reloading (re-activation of physical activity). Thus, re-starting physical activity in low-activity patients may bring early results that can be effective for preventing complications associated with diabetic lipid metabolism. Early improvements in the lipid profile associated with such re-activity may reinforce the usefulness of rehabilitation. However, the present study was associated with some limitations. It is unclear whether the results are applicable in humans, and some measurements were lacking, including physical activity after animal reloading, insulin resistance, the muscle protein synthesis function, and the IMLDs of medial Gas muscle.

## Conclusions

Although no marked differences in the skeletal muscle protein components or IMLD concentrations were noted, we concluded that activity-dependent changes in HDL cho in relation to total cho might be more sensitive in T2DM rats than non-DM rats, especially in early stages.

## Supplementary Information

**Additional file 1.**

## Data Availability

All data generated or analyzed during this study are included in this published article.

## Abbreviations

BW: body weight
cho: cholesterol
CON: control
DM: diabetes mellitus
Gas: gastrocnemius
GK: Goto-Kakizaki
HDL: high-density lipoprotein
IMLD: intramuscular lipid droplet
LDL: low-density lipoprotein
MCP: muscle component protein
MCP mass: muscle dry weight per body weight
muscle mass: muscle wet weight per body weight
MCP ratio: muscle dry weight per muscle wet weight; non-DM: non-diabetes mellitus
ORO: oil red O
PBS: phosphate buffered saline
R1w: groups reloaded for 2 weeks following tail suspension
R2w: groups reloaded for 2 weeks following tail suspension
RT: room temperture
SDS: sodium dodecyl sulfate
T2DM: type 2 diabetes mellitus
