# Supplementary figures and images for: Blood lipid profile changes in type 2 diabetic rats after tail suspension and reloading
Source: Lipids Health Dis. 2021 Aug 1;20:84. doi: 10.1186/s12944-021-01511-y (PMC8327430; doi:10.1186/s12944-021-01511-y)

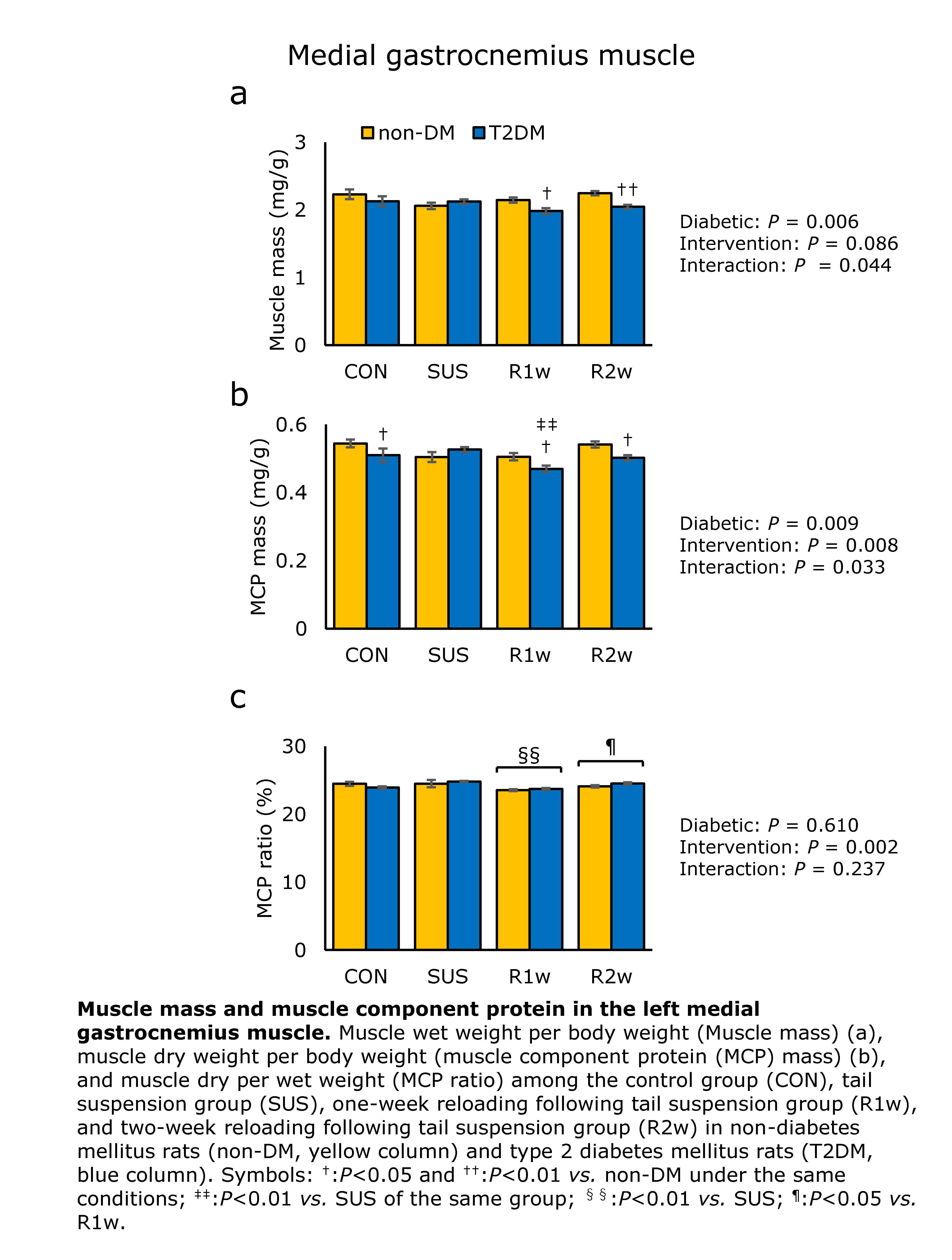

Supplement: Supplementary file 1 — Additional file 1. [file 12944_2021_1511_MOESM1_ESM.jpg]
